# Examining the Inheritance of Watermelon Fruit Traits by Hayman's Graphical Approach

**DOI:** 10.1155/2022/3059218

**Published:** 2022-02-27

**Authors:** Mehdi Rahimi, Maryam Abdolinasab

**Affiliations:** Department of Biotechnology, Institute of Science and High Technology and Environmental Sciences, Graduate University of Advanced Technology, Kerman, Iran

## Abstract

Watermelon traits such as yield and other agronomic traits are highly environmentally sensitive and regulated by multiple genes; therefore, by understanding the genetic structure, the heritability and genetic influence of different traits can improve them. Five watermelon lines were crossed in a 5 × 5 full diallel parental design to estimate the genetic effect and heritability of fruit traits. Treatments were evaluated on the farm using a random complete block design. Analysis of the results showed a significant difference between genotypes, which was observed for all the studied traits at the probability level of 1%. Hayman's graphical method showed that the contribution of the nonadditive effects was more important than that of the additive effect to control most of the traits. Fruit maturation and pericarp thickness traits were regulated by incomplete dominance gene effects, and other traits were regulated by overdominance effects. The trait heritability varied between at least 0.013 and 0.352 for the fruit weight and fruit number, respectively. Results demonstrated that some traits can be modified based on the heterozygosity and production of hybrid variety methods, while the hybrid and selection in an advanced generation method can be suggested in watermelon breeding programs to breed other traits.

## 1. Introduction

The family Cucurbitaceae has different species, and the only cultivated species of this genus is watermelon (*Citrullus lanatus* var. Lanatus) with 2n = 2x = 22 chromosomes [1]. Watermelon originates from the South African region [2], and watermelon is currently being cultivated worldwide as a fruit crop. China has taken the first place with 62,803,768 tons (60.40% production) and Iran (4,113,711 tons); Turkey (4,031,174 tons), India (2,520,000 tons), and Brazil (2,240,796 tons) are in the next places [3]. Different species and varieties of watermelon are very similar at the beginning of growth but show great variation in fruit shape and other traits [4]. Therefore, breeding programs to produce a new cultivar need more information about the genetic components as well as the type of gene action of traits in order to increase yield and produce tolerant cultivars [5–7]. Agronomic and yield traits of watermelon are highly environmentally sensitive and regulated by multiple genes, making them quantitatively complex traits with low inheritance. Thus, by understanding the genetic structure, the heritability and genetic influence of different traits can improve yield [8].

The first step in the plant breeding programs is to study genetic diversity, as was performed by many studies on the genetic diversity of watermelon. For example, Mohosina et al. [9] divided 16 watermelon hybrids into 5 different groups and used them in breeding programs. Also in the research of Pratami et al. [10], the ISSR marker was used to study the genetic diversity of Cucumis and Mukia (*Cucurbitaceae*). Then, the application of these results in other studies, for example, in the research by Wu et al. [11], expressed the understanding of the relationship between lycopene *β*-cyclase (lcyb) protein sequences and watermelon flesh color, and in another study by Reddy et al. [12], the correlation between yield and biochemical traits was examined in snapmelon (*Cucumis melo* var. momordica).

In plant breeding, to achieve cultivars with desirable agronomic traits, knowledge of the genetic structure of the crossed parents is very important for the researcher to choose the appropriate breeding method. Knowledge about how the inheritance and type of action of genes controlling the traits is the basis of designing a suitable breeding method to achieve the goals of genetic breeding. One of the methods, by which genetic information can be obtained easily and in a relatively short time, is the method of diallel crosses; the principles and foundations of which were presented by Jenks, Hayman, and Griffin in the 1950s [13–21]. Among the important parameters that are estimated by this method are the amount of heterosis, type of gene action, and the general and specific combining ability of breeding lines [8].

Diallel analysis has been used as a suitable and efficient method for quantitative trait genetic analysis to estimate the genetic structure and genetic effects of agronomic traits considered by breeders [8]. Among the various methods of diallel analysis, Hayman's graphic method which is based on estimating variance components provides useful information about the genetic parameters of quantitative traits and the genetic status of the studied parents. These components include (a) additive variance and (b) nonadditive variance. Also, through Hayman's graphic method, it is possible to easily show the type of gene action (additive effect, complete or incomplete dominance effects, and overdominance gene action) in controlling the studied trait [22]. The diallel analysis through Hayman's graphical approach was first developed by these scientists [15–21].

Several studies based on the Griffing diallel method had been done on watermelon to estimate combining ability [5, 6, 23–28]. The Griffing method is also used to estimate heterosis and identify the superior hybrid, such as studies by Moon et al. [29] on the muskmelon that identified the *F*_1_ hybrid *P*_2_ × *P*_8_ (Pusa Madhuras×Hara Madhu), as the superior hybrid. In addition, these studies show that inheritance and control of the studied traits are equally affected in both additive and nonadditive effects. However, in the study by Sapovadiya [26] based on Hayman's diallel method of eight watermelon varieties for fruit weight, fruit yield, flesh weight, and number of fruits per plant, it was demonstrated that the role of nonadditive effects in controlling them was greater than that of additive effects.

Although diallel crosses have been studied to investigate the combing ability (GCA and SCA) of different traits of crops, few works have been done on watermelon, especially on how to inherit its quantitative traits in the form of diallel crosses. Also, the type of gene action (additive, dominance, and overdominance effects) in controlling fruit traits in watermelon by the Hayman method has not been done much. Therefore, for better planning and selection of breeding methods according to the type of genes in controlling watermelon traits, a 5 × 5 diallel design was conducted. Therefore, the purpose of this study was to investigate the effect of genes, heritability, and control of fruit traits in watermelon in order to select appropriate breeding methods and take an effective step in producing high-yielding watermelon cultivars.

## 2. Material and Methods

This study was performed on five accessions of *Citrullus lanatus* plants (Orzoeiyeh (P1), Hejrak (P2), Gerd (P3), Neyshabour (P4), and Yazd (P5)) from plant materials collected from different parts of Iran. Firstly, to select these five parents, 38 watermelon genotypes that were collected from different regions and gene banks were planted in a statistical design and different traits were measured and their diversity was estimated by cluster analysis. Finally, based on the greatest genetic distance in terms of different traits and cluster analysis results, popular cultivars were selected from each group and used in this study, and these five parents were the most popular cultivated genotypes in the population.

Prior to crossing to obtain different *F*_1_ families, the purity of the five parent accessions was achieved after several generations of self-pollination. Afterwards, from April to May 2017, seeds of 5 parental lines were sown for crossing all the parents together based on the diallel design; it produced 20 *F*_1_ hybrids with 5 inbreeding lines. A randomized full block design (RCBD) with three replicates was used in this study. Therefore, the *F*_1_ offspring and their parents were planted in the research farm based on this design from May to October 2017 at the Graduate University of Advanced Technology, Kerman. This research farm was located at the Graduate University of Advanced Technology with E 57° 17′ longitude and N 30° 1′ latitude and with the altitude of 2020 m above sea level. Climatic characteristics of this region were hot and dry climates. The average annual temperature was 16 degrees, average annual rainfall was 136 mm, and average humidity was 31% [30]. The row-to-row and plant-to-plant distances were kept at 2 m and 0.5 m, respectively. During the growing season, data for various morphological and agronomic traits were measured based on the descriptors [31]. Ten ripe fruits of each genotype per plot and six individual plants of each genotype per plot were individually sampled to measure the fruit and agronomic traits, respectively. Finally, the means of each genotype were used in the analyses. Traits included flesh weight, skin thickness, fruit number per plant, fruit maturity period, fruit weight/plant, pH, fruit length/plant, and sugar content. Sugar content, determined as total soluble solids, was measured as Brix using a handheld digital refractometer [32].

After the analysis of variance, the data were tested using an additive dominance model, which required calculating the variance (*V*_*r*_) of each array component and the covariance of the array's parent offspring (*W*_*r*_). Scaling tests were performed to verify the fit of the additive-dominant model to the specified data using regression analysis and variance analysis for arrays (*W*_*r*_ + *V*_*r*_ and *W*_*r*_ − *V*_*r*_) and *t*^2^ tests. Hayman's analysis method shows the additive and dominant effects related to the inheritance of the studied traits based on the analysis of variance and covariance, and by using this method [15–19], the mean degree of dominance, presence of nonallelic interaction, degree of heritability, and the ratio and distribution of alleles in parents can be examined. The parameters and statistical indicators that were calculated in this model are as follows: *V*_p_: parents' variance; *W*_*r*_: parents' and their offspring covariance in the *r*th row; $\overset{¯}{W_{r}}$: mean covariance of parents and their offspring in rows; *V*_*r*_: variance of row *r*; $\overset{¯}{V_{r}}$: mean of row variance; $V_{\overset{¯}{r}}$: variance of row means; *m*_p_: mean of parents; and *m*_o_: mean of offspring.

Drawing the regression line *W*_*r*_ on *V*_*r*_, determining the coordinates of the parent location, and plotting the parabola plot are the tests for approving the validity of diallel hypotheses and demonstrate the complete dominance of the genes, in which case it is *h* = *d*, if the regression line passes through the center of coordinates (intercepts equal to zero or *a* = 0). If the line intersects the *W*_*r*_ axis in the positive part (i.e., *a* > 0 and *h* < *d*), there will be a reason for the effect of incomplete dominance of genes. Also, if this line intersects the *W*_*r*_ axis in the negative part, i.e., it is *a* < 0, therefore, *h* > *d*; it is a reason for the overdominance gene effect. When the regression line is tangential to the parabola plot, it is a sign of the lack of dominance and, consequently, the additive effect of genes. For different degrees of dominance, the dominant homozygous parent is always at the bottom and the recessive homozygous parent is always at the top of the regression line [15–19].

The total phenotypic variance was divided into its environmentally induced part *E*, and five different genetic components that have been defined by Jinks and Hayman and were estimated as follows [15–19]: *D*: variations due to an additive effect; *H*_1_: the variation components due to the effect of gene dominance; *H*_2_: calculations to predict the proportion of positive and negative genes in parents; *F*: mean of Fr values over arrays, where Fr is the covariance of additive and dominance effects in a single array and *F* is positive where dominant genes are more frequent than recessive; *E*: expected environmental component of variation; and *h*: dominance effects (as algebraic sum on all heterozygous loci in all crosses. Positive *h* indicates the predominance of genes with capital letters, and negative *h* indicates the predominance of genes with lower letters. In both cases, the dominant genes are below the regression line and the recessive genes are above the regression line).

The relationship between variances, covariances, and genetic parameters is as follows:

(1) $$\begin{array}{c} V_{P}=V_{p}=D+E,\\ W_{r}=\frac{1}{2}D-\frac{1}{4}F_{r}+\frac{E}{n},\\ {\overset{¯}{W}}_{r}=\overset{¯}{W_{r}}=\frac{1}{2}D-\frac{1}{4}F+\frac{E}{n},\\ V_{r}=\frac{1}{4}D-\frac{1}{4}F_{r}+\frac{1}{4}H_{1}+\frac{E}{n},\\ {\overset{¯}{V}}_{r}=\frac{1}{4}D-\frac{1}{4}F+\frac{1}{4}H_{1}+\frac{E}{n},\\ V_{\overset{¯}{r}}=\frac{1}{4}D-\frac{1}{4}F+\frac{1}{4}H_{1}-\frac{1}{4}H_{2}+\frac{E}{n^{2}},\\ {\left(m_{O}-m_{P}\right)}^{2}=\frac{1}{4}h^{2}+\frac{E}{n^{3}}\left(n-1\right),\\ E=\frac{\left[\left(\text{error }S.S.+\text{reps}.S.S.\right)/d.f.\left(\text{error}\right)\right]}{\text{number of replications}},\\ \overset{\wedge }{D}=V_{P}-\overset{\wedge }{E}{\overset{\wedge }{F}}_{r}=2\left(V_{P}-{\overset{¯}{W}}_{r}+{\overset{¯}{V}}_{r}-W_{r}-V_{r}\right)-\frac{2\left(n-2\right)\overset{\wedge }{E}}{n},\\ {\overset{\wedge }{H}}_{2}=4{\overset{¯}{V}}_{r}-4V_{\overset{¯}{r}}-2\overset{\wedge }{E}{\overset{\wedge }{H}}_{1}=V_{P}-4{\overset{¯}{W}}_{r}+4{\overset{¯}{V}}_{r}-\frac{\left(3n-2\right)\overset{\wedge }{E}}{n},\\ \overset{\wedge }{F}=2V_{P}-4{\overset{¯}{W}}_{r}-\frac{2\left(n-2\right)\overset{\wedge }{E}}{n},\\ \overset{\wedge }{h^{2}}=4{\left(m_{O}-m_{P}\right)}^{2}-\frac{4\left(n-1\right)\overset{\wedge }{E}}{n^{2}},\\ n=d.f.\left(\text{error}\right)\times \text{number of replications}. \end{array}$$

Parental and *F*_1_ generation results were analyzed according to Hayman's method, and graphical analysis was performed using it [19]. Genetic parameters including additive variance (*D*), nonadditive variance (*H*_1_ and *H*_2_), and covariance of additive effects with dominance (*F*) were also estimated by the proposed Hayman's regression method [16, 19]. Then, the following genetic ratios were determined based on these estimates.

The $\sqrt{H_{1}/D}$ ratio was used for showing the average degree of dominance of the loci controlling each trait [16, 19]. *H*_2_/4*H*_1_ is the proportion of genes with positive and negative effects in parents. *h*^2^/*H*_2_ denotes the number of gene groups/genes, which control the character and exhibit dominance.$\;\sqrt{4DH_{1}+F}/\sqrt{4DH_{1}-F}$ denotes the ratio of dominant and recessive genes in the parents; if the ratio is 1, the dominant and recessive genes in the parents are in equal proportion; if it is less than 1, it indicates an excess of recessive genes; but being greater than 1, it indicates excess of dominant genes.

In the *F*_1_ generation, the broad and narrow sense heritability values were calculated for each trait from the following relationship [16, 19]:

The broad sense heritability (*F*_1_) or *h*_*b*_^2^ is

(2) $$\begin{array}{c} h_{n}^{2}=\frac{\left(1/2\right)D+\left(1/2\right)H_{1}-\left(1/2\right)H_{2}-\left(1/2\right)F}{\left(1/2\right)D+\left(1/2\right)H_{1}-\left(1/2\right)H_{2}-\left(1/2\right)F+E}. \end{array}$$

The narrow sense heritability (*F*_1_) or *h*_*n*_^2^ is

(3) $$\begin{array}{c} h_{n}^{2}=\frac{\left(1/2\right)D+\left(1/2\right)H_{1}-\left(1/2\right)H_{2}-\left(1/2\right)F}{\left(1/2\right)D+\left(1/2\right)H_{1}-\left(1/2\right)H_{2}-\left(1/2\right)F+E}. \end{array}$$

Also, other indices and parameters such as the dominant and recessive gene ratio, direction of dominance gene effects (the correlation coefficient of *W*_*r*_ + *V*_*r*_ and the parent mean), and positive and negative effects on the parent ratio were calculated based on Hayman's proposed methods [19]. The regression coefficient of *W*_*r*_ on *V*_*r*_ was used for epistasis and the hypothesis of Hayman's method test [16]. The macroprogramming designed in SAS software was used to perform the diallel analysis based on Hayman's method [33]. The SASHAYDIALL program was written in SAS/IML by Makumbi et al. [33], and this code as well as the sample data can be downloaded at the following link: https://data.cimmyt.org/dataset.xhtml?persistentId=hdl:11529/10548045.

## 3. Results and Discussion

The simple effect of the genotype was broken down into components *a*, *b* (*b*1, *b*2, and *b*3), *c*, and *d* (Table 1), and these effects represent the additive genetic effects, dominant genetic effects, average maternal effects of each parental line, and variation in the reciprocal differences not attributed to *c*, respectively. The two tests were performed for each of the simple components, and this effect can be tested on the interaction effects of each component×replication and experiment error (Table 1).

Furthermore, the preliminary test results of the Jinks-Hayman model for the slope of the regression line *W*_*r*_ on the *V*_*r*_ (*t*^2^), the *W*_*r*_ − *V*_*r*_ test, and the genetic components of variances and other statistical indices, viz., *D*, *H*_1_, *H*_2_, *h*^2^, *E*, and *F*, for the studied traits are shown in Table 2. The results of the Jinks-Hayman preliminary test showed no epistatic effects of genes on controlling studied traits, and diallel graphical analysis could be completely performed for them (Table 2).

The results of Hayman's variance analysis (Table 1) showed that genotypes (parents and offspring) had significant differences in yield and fruit traits. The role of additive effects was determined due to the significance of “*a*” effect in controlling all traits, except the fruit weight per plant, flesh weight, and skin thickness (Table 1). Moreover, the role of nonadditive effects was shown for controlling all the traits (Table 1) due to the significance of “*b*” effect (except the fruit number per plant). Based on the significance of the “*b*_1_” effect, it was found that there was a difference between the parents and progeny for all the traits which showed the directional dominance for traits, except for the fruit number per plant, pH, and sugar content. Although, the study by Badami et al. [34] based on Griffing's method revealed that the traits of harvest age, fruit flesh thickness, fruit total soluble solids, fruit length, and fruit weight were controlled by dominant gene action, whereas the fruit diameter was managed by additive and dominant genes.

The dominant and recessive gene frequency in parents was not the same for all the traits, and the significance of the “*b*_2_” effect (Table 1) determined it, while it was equal for the fruit number per plant and fruit length traits. Also, the same frequency of these genes in parents was determined when this effect was not significant. In addition, the significance of specific combining ability (Table 1) was determined based on the significance of the “*b*_3_” effect for the studied traits, except the fruit number per plant. The simple effect “*c*” indicating maternal effects was significant for all the traits (except for skin thickness and pH traits), and the “*d*” effect showed simple reverse effects and was significant for the studied traits, except for the fruit number per plant and pH traits (Table 1). Therefore, the role of maternal and reverse effects was identified in controlling them (significant traits for “*c*” and “*d*” effects, respectively).

Estimates of statistical indices and genetic components for the studied traits are presented in Table 2. The significance of parameter *D* showed that additive effects were involved in controlling traits. This parameter was significant for the fruit number per plant, fruit weight per plant, fruit length, flesh weight, and sugar content traits, showing the role of additive effects in controlling them. The significance of *H*_1_ and *H*_2_ parameters also demonstrated the role of dominance effects in controlling the traits. Considering the significance of these parameters for the fruit maturity period, fruit weight per plant, fruit length, flesh weight, skin thickness, pH, and sugar content traits, the role of the dominance effect in the inheritance of them was determined.

The significance of *D*, *H*_1_, and *H*_2_ parameters showed the role of simultaneous additive and nonadditive effects in controlling traits that were significant for fruit weight per plant, fruit length, flesh weight, skin thickness, pH, and sugar content traits. But the other traits were only controlled by nonadditive effects. Studies by other researchers have reported similar and different results for the type of genes controlling these traits [5, 6, 26, 27], which may be due to the type of parent, allele's distribution in the parent, and the interactions between the environment and genes of traits.

The *H*_2_/4*H*_1_ ratio, which shows an estimate of the ratio of dominant genes with increasing (positive) to decreasing (negative) effects, in the best case, i.e., in the symmetry of gene frequencies in parents, will be 0.25. Otherwise, it indicates the asymmetry of the dominant positive and negative alleles of the genes controlling the relevant traits in the parents. In other words, the farther this ratio is from 0.25, asymmetric and nonuniform distributions of positive and negative dominant alleles will show more in parents. These ratios were 0.165, 0.19, 0.23, 0.24, 0.22, 0.20, 0.21, and 0.16 for the fruit number per plant, fruit maturity period, fruit weight per plant, fruit length, flesh weight, skin thickness, pH, and sugar content, respectively, indicating that the dominant additive and subtractive genes in the parents were different for all the traits. The overdominance effects played a role in controlling all the traits, except the fruit number per plant, which was due to the point that the mean degree of dominance $\left(\sqrt{H_{1}/D}\right)$ was higher than 1 for those traits like the results of graphical Hayman's analysis. Estimations of narrow sense heritability based on the Hayman-Jinks model for the fruit number per plant, fruit maturity period, fruit weight per plant, fruit length, flesh weight, skin thickness, pH, and sugar content were 0.352, 0.345, 0.013, 0.195, 0.038, 0.079, 0.226, and 0.151, respectively, indicating low heritability of these traits. Therefore, given the high contribution of nonadditive effects of genes on controlling these traits, selection potential for these traits would not be high; therefore, hybridization- and selection-based breeding methods in advanced generations may be useful (Zare et al., 2011).

The *r* parameter, which shows the correlation between parents and *W*_*r*_ + *V*_*r*_, indicates the behavior of dominant alleles. If it is negative, it indicates that the dominant alleles are subtractive and these alleles decrease the trait. Also, if it is positive, it indicates an additive effect of dominant alleles and these alleles increase the trait. In this study, the *r* parameter or correlation coefficient (Table 2) was negative for the studied traits (fruit number per plant, fruit weight per plant, fruit length, flesh weight, and skin thickness), indicating that the dominant alleles for this trait were subtractive and reducing the trait. The $\sqrt{4DH_{1}+F}/\sqrt{4DH_{1}-F}$ ratio shows the ratio of dominant and recessive alleles in the parents, so that when this ratio is equal to one, the dominant and recessive genes in the parents are equal. When this ratio is less than one, it indicates the greater frequency of recessive genes in parents. When this ratio is more than one, the dominant genes are more in the parents. Calculating the $\sqrt{4DH_{1}+F}/\sqrt{4DH_{1}-F}$ ratio also showed that all the studied lines had more dominant alleles than the recessive ones for all the studied traits.

**Figure 1:**
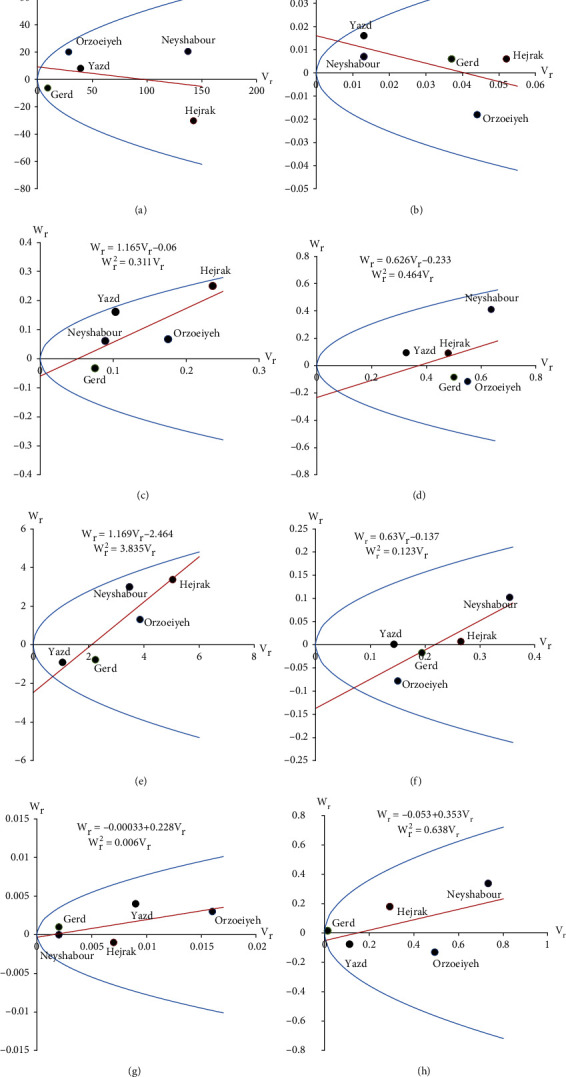
*W*_*r*_/*V*_*r*_ graph for the studied traits of watermelon. (a) Fruit maturity period, (b) skin thickness, (c) fruit number/plant, (d) fruit weight/plant, (e) fruit length, (f) flesh weight, (g) pH, and (h) sugar content.

Parental distribution for the studied traits is shown in Figures 1(a)–1(h). According to the *W*_*r*_ regression line on *V*_*r*_, the parents that were closest to the *W*_*r*_ axis regression line had the highest number of dominant genes and the parents that had the farthest distance from the *W*_*r*_ axis regression line had the most recessive alleles. The distribution of parent for the fruit number per plant (Figure 1(a)) and skin thickness (Figure 1(b)) showed that these traits were controlled by the dominance gene effects; therefore, the breeding method of these traits was hybridization and selection in advanced generations.

Also, the regression line *W*_*r*_ on *V*_*r*_ was cut in the negative part of the *W*_*r*_ axis for other traits (Figures 1(c)–1(h)), meaning that these traits were affected by the overdominance effect of genes. Since these traits were controlled by overdominance gene effects, the heterosis phenomenon can be exploited to increase and improve these traits. The distribution of parents along the regression line showed that Yazd and Gerd cultivars for traits of the fruit number/plant, fruit weight/plant, fruit length, and flesh weight were the closest parents to the point where the regression line collided with the *W*_*r*_ axis; therefore, these cultivars had the maximum number of dominant genes. Due to the reduction of the dominant gods for these traits (negative *r* for these traits), these parents cannot be used to increase this trait in breeding programs. According to the results of this experiment, which showed that genetic control of these traits was under the influence of the overdominance gene effects, breeding methods based on hybridization and heterosis phenomenon should be used to improve these traits in breeding programs. Due to the different patterns of gene expression from one environment to another, different strategies should be used to modify these traits in different environmental conditions.

## 4. Conclusions

The role of overdominance and incomplete dominance effects in controlling studied traits was identified in this study. According to the results, the genetic control of the traits was different; therefore, a specific breeding strategy should be used for each trait. In most of the traits studied, genes with the overdominance effects had a dominant role in controlling the traits. In addition, the estimation of private heritability of traits indicated that most of the studied traits were of moderate private heritability and nonadditive effects played a greater role in controlling them. Hence, the direct selection breeding method would not be very successful in improving the genetic value of the population for these traits, but using the heterosis phenomenon and the crossing the parents to produce hybrids or direct selection of lines in the last generations could improve these traits.

## Figures and Tables

**Table 1:** Variance analysis of different traits of watermelon using Hayman's method.

Mean square of studied traits								DF	Source of variation
Sugar content	pH	Skin thickness	Flesh weight	Fruit length	Fruit weight/plant	Fruit maturity period	Fruit number/plant		
0.696^ns^	0.006^ns^	0.031^ns^	0.14^ns^	1.94^ns^	0.651^ns^	3.04^ns^	1.013^ns^	2	Replication
1.37^∗∗##^	0.026^∗∗##^	0.139^∗∗##^	0.845^∗∗##^	14.60^∗∗##^	2.117^∗∗##^	231.97^∗∗##^	1.102^∗∗##^	24	Genotype
1.38^#^	0.04^∗∗##^	0.077^ns^	0.316^ns^	14.99^∗##^	0.64^ns^	449.86^∗∗##^	1.57^##^	4	*a*
1.69^∗∗##^	0.036^∗∗##^	0.18^∗∗##^	1.26^∗∗##^	15.81^∗∗##^	2.86^∗∗##^	337.98^∗∗##^	0.49^ns^	10	*b*
0.32^ns^	0.008^ns^	0.49^∗∗#^	4.95^∗##^	80.94^∗∗^	8.81^##^	24.65^##^	0.21^ns^	1	*b*_1_
2.85^∗##^	0.028^∗∗#^	0.14^∗##^	0.77^∗∗##^	5.20^ns^	1.18^∗∗^	379.93^∗∗##^	0.58^ns^	4	*b*_2_
1.05^∗#^	0.048^∗∗##^	0.139^∗∗##^	0.93^∗##^	11.27^∗#^	3.02^∗##^	367.09^∗∗##^	0.49^ns^	5	*b*_3_
1.05^∗^	0.013^ns^	0.027^ns^	0.48^#^	12.22^#^	1.42^#^	58.63^∗∗##^	2.7^∗∗##^	4	*c*
1.03^#^	0.008^ns^	0.196^∗∗##^	0.74^##^	13.92^##^	2.32^∗##^	25.58^∗∗##^	0.73^ns^	6	*d*
0.42	0.008	0.032	0.175	3.43	0.519	1.97	0.354	48	Error

ns: nonsignificant; ^∗^significant at 5% probability level; ^∗∗^significant at 1% probability level (each of the terms was tested against the interaction of each term with replication); ^#^significant at 5% probability level; ^##^significant at 1% probability level (all terms were tested against the experimental error).

**Table 2:** Genetic parameters of different traits in watermelon based on Hayman's method.

Sugar content	pH	Skin thickness	Flesh weight	Fruit length	Fruit weight per plant	Fruit maturity period	Fruit number per plant	Genetic parameter^#^
0.14^ns^	0.0027^ns^	0.011^ns^	0.058^∗∗^	1.12^∗∗^	0.17^∗∗^	0.67^ns^	0.127^∗∗^	*E*
0.49^∗^	0.0029^ns^	0.02^ns^	0.065^∗^	2.71^∗∗^	0.29^∗^	24.96^ns^	0.184^∗∗^	*D*
0.85^ns^	0.0026^ns^	0.04^ns^	0.165^ns^	1.53^ns^	0.40^ns^	40.83^ns^	0.066^ns^	*F*
1.32^∗^	0.023^∗^	0.12^∗^	0.85^∗∗^	8.66^∗∗^	1.69^∗∗^	299.56^∗^	0.119^ns^	*H*_1_
0.84^∗^	0.019^∗^	0.095^ns^	0.73^∗∗^	8.29^∗∗^	1.56^∗∗^	223.98^∗^	0.079^ns^	*H*_2_
0.00001^ns^	0.000001^ns^	0.098^∗^	1.02^∗∗^	16.55^∗∗^	1.77^∗∗^	4.83^ns^	0.0001^ns^	*h*
1.64	2.77	2.35	3.60	1.79	2.42	3.46	0.805	$\sqrt{\frac{H_{1}}{D}}$
0.16	0.21	0.20	0.22	0.24	0.23	0.19	0.165	$\frac{H_{2}}{4H_{1}}$
3.22	1.39	2.18	2.09	1.37	1.81	1.62	1.57	$\frac{\sqrt{4DH_{1}+F}}{\sqrt{4DH_{1}-F}}$
0.50	0.28	−0.55	−0.46	−0.95	−0.37	0.098	−0.797	*r*
0.000001	0.00014	1.03	1.40	1.99	1.14	0.022	0.0001	$\frac{h^{2}}{H_{2}}$
0.151	0.226	0.079	0.038	0.195	0.013	0.345	0.352	*h*_*n*_^2^
0.655	0.717	0.715	0.768	0.717	0.694	0.992	0.439	*h*_*b*_^2^
−0.053	−0.00033	0.016	−0.137	−2.464	-0.233	9.16	−0.057	*a*
0.284^ns^	0.518^ns^	0.518^ns^	0.138^ns^	−0.481^ns^	1.42^ns^	5.7^ns^	1.48^ns^	*t* ^2^
0.000005^ns^	0.0076^ns^	0.0076^ns^	0.02^ns^	0.93^ns^	0.05^ns^	3711.68^ns^	0.027^ns^	*W*_*r*_ − *V*_*r*_

ns: nonsignificant; ^∗^significant at 5% probability level; ^∗∗^significant at 1% probability level; ^#^*E*: environmental variance; *D*: additive variance; *F*: covariance of additive with dominance effect; *H*_1_ and *H*_2_: dominance variances; *h*: dominance effect over all loci in the heterozygous phase; $\sqrt{H_{1}/D}$: mean degree of dominance; *H*_2_/4*H*_1_: proportion of dominance genes with increasing and decreasing effects; $\left(\sqrt{4DH_{1}}+F\right)/\left(\sqrt{4DH_{1}}-F\right)$: proportion of all genes with positive and negative effects in the parents; *r* correlation between parent means (*Y*_*r*_) with ${\underset{¯}{W}}_{\underset{¯}{r}}+V_{r}$; *h*/*H*_2_: number of gene blocks controlling the trait and exhibiting dominance; *h*_*n*_^2^: narrow sense heritability; *h*_*b*_^2^: broad sense heritability; *a*: intercept of regression line; *t*^2^: significant test of regression coefficient from one; *W*_*r*_ − *V*_*r*_ analysis of variance for *W*_*r*_ − *V*_*r*_ over replications.

## Data Availability

The [Supplementary File 1] data used to support the findings of this study are included within the supplementary information file.

## Supplementary Materials

Supplementary Materials This file is about the raw data of the eight studied traits that were measured on the offspring and parents (all the parents crossed together based on the diallel design and produced 20 F1 hybrids with 5 inbreeding lines) based on a randomized complete block design with three replications in three study environments.
